# Supplementary material for: “Candidatus Campylobacter infans” detection is not associated with diarrhea in children under the age of 2 in Peru
Source: PLoS Negl Trop Dis. 2022 Oct 17;16(10):e0010869. doi: 10.1371/journal.pntd.0010869 (PMC9612815; doi:10.1371/journal.pntd.0010869)
Supplement: S1 Table — (DOCX) [file pntd.0010869.s001.docx]

**S1Table.** gBlock Gene Fragments sequences used as positive controls

| **Target** | **Sequence** |
| --- | --- |
| 16s control | AGT TGC TAA CAG TTT GGC TGA GCA CTC TAA ATA TAC TGC CTT CGT AAG GAG GAG GAA GGT GTG GAC GAC GTC AAG TCA TCA TGG CCC TTA TGC CCA GGG CGA CAC ACG TGC TAC AAT GGC ATA TAC AAT GAG ACG CAA TAT CGC GAG ATG GAG CAA ATC TAT AAA ATA TGT CCC AGT TCG GAT TGT TCT CTG CAA CTC GAG AGC ATG AAG CCG GAA TCG CTA GTA ATC GTA GAT CAG CCA TGC TAC GGT GAA |
| cadF control | TAT CAT AAG CAG CAT TTG AAA AAT CCT CAT ATC CTC CAC CTG CTA AAC CAT AGA AAT AAA ATT TCT CAC CTA CAT CAA TAC CTT TAA TAG CAC TCA AAT AAG TTC TTG TAA TAT CTG TAG TTT TAT TTG TAT TTG TAT ATT TAA CAT CAG AAT AAT GCT CTA ACC CAA ATT CTA ATT GAT CAA GCC AAA AAT CGT CAA AAT GAT AAC CAA GTC TAA TCC CTG GTG CAT AAC GAT TAT CCA TAT CTA AAT TAC CTT CAA AGG ACT GAT GCT CAG TGA GTT ACT ACG CAG TCA CTC A |
| ipaH control | GAA TAC ACT CCA TCG CCC CCT GGC TGA TGC CGT GAC AGC ATG GTT CCC GGA AAA CAA ACA ATC TGA TGT ATC ACA GAT ATG GCA TGC TTT TGA ACA TGA AGA GCA TGC CAA CAC CTT TTC CGC GTT CCT TGA CCG CCT TTC CGA TAC CGT CTC TGC ACG CAA TAC CTC CGG ATT CCG TGA ACA GGT CGC TGC ATG GCT GGA AAA ACT CAG TGC CTC TGC GGA GCT TCG ACA GCA GTC TTT CGC TGT TGC TGC |
| Infans (*lpxA)* control | GGC CAT TAT AAA AGC ATT ATC GCC TAT TAG CGT GTA ACC ATC GCA TTC ATC GTT TTT ATG CGT GCC TGA GTT TAT CGT GCA AAA CTC CCT TAT GCT AGC ATT TTT GCC TAT TAC TAC CCC GCA ACC CTC GCC ACT GCT TTT TGC ATA GCT TAT GTC TTG GCA AAT ATC GCC TAT GAT GGC GTA GCT ATA AAT TAT GGA ATT TTG GCC TAT TTT TGT GTT GCC GAC TAT TCT TGC GCC TTG TTT TAT GGT GCA ATT TTG CTC AAT TAC GGC ATT TTT ACC CAC AAA G |
