# Supplementary material for: “Candidatus Campylobacter infans” detection is not associated with diarrhea in children under the age of 2 in Peru
Source: PLoS Negl Trop Dis. 2022 Oct 17;16(10):e0010869. doi: 10.1371/journal.pntd.0010869 (PMC9612815; doi:10.1371/journal.pntd.0010869)
Supplement: S1 Fig — Box and whiskers blot showing no statistical differences in the quantity of (Log10) of each qPCR target between symptomatic and asymptomatic samples. Quantitative data shown below: (DOCX) [file pntd.0010869.s003.docx]

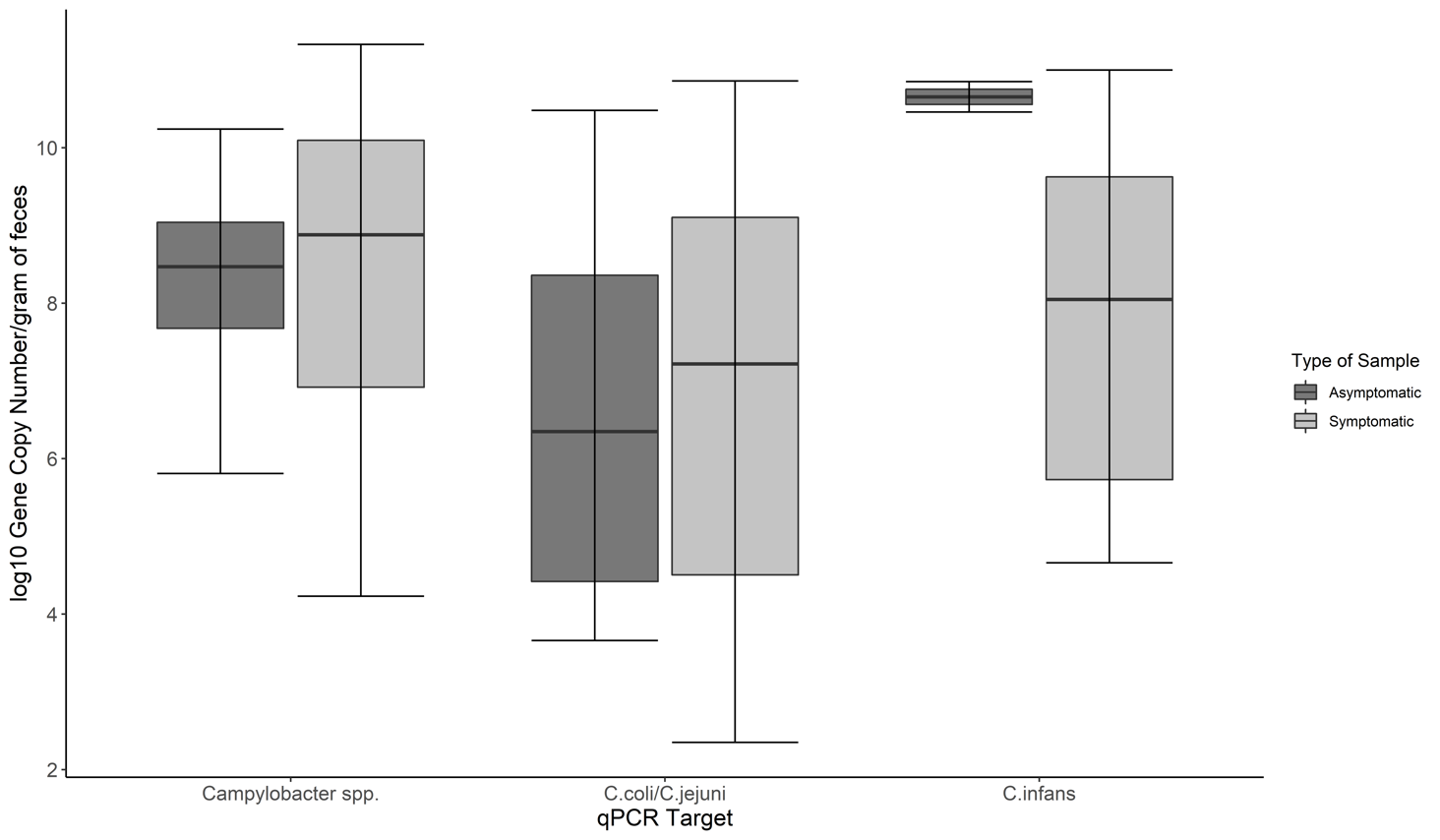
**S1 Fig.** Gene Copy Number (Log10) of qPCR Targets Per Gram of Feces in Symptomatic and Asymptomatic Fecal Samples.

Box and whiskers blot showing no statistical differences in the quantity of (Log10) of each qPCR target between symptomatic and asymptomatic samples. Quantitative data shown below:

| Target Species | Mean (log10) Gene Copy Number per Gram of Feces | | |
| --- | --- | --- | --- |
|  | Asymptomatic | Symptomatic | p-value* |
| *Campylobacter* spp. (16S rRNA) | 6.60 | 6.86 | 0.651 |
| *C. jejuni / C. coli (cadF)* | 8.25 | 8.37 | 0.912 |
| *C. infans (lpxA)* | 10.66 | 7.76 | 0.077 |
| *Independent Two-sample t-test |  |  |  |
