# Supplementary material for: “Candidatus Campylobacter infans” detection is not associated with diarrhea in children under the age of 2 in Peru
Source: PLoS Negl Trop Dis. 2022 Oct 17;16(10):e0010869. doi: 10.1371/journal.pntd.0010869 (PMC9612815; doi:10.1371/journal.pntd.0010869)
Supplement: Attachment — Submitted filename: Cinfans_manuscript_2022_ReviewersResponse_20sept.docx [file pntd.0010869.s005.docx]

September 20, 2022

Dr Shaden Kamhawi, Dr Paul Brindley, and Dr Peter Hotez

Editors-in-Chief

*PLOS Neglected Tropical Diseases*

**REF: PNTD-D-22-00633**

Dear Dr Shaden Kamhawi, Dr Paul Brindley, and Dr Peter Hotez,

I would like to thank you for the review of our "*Prevalence of “Candidatus Campylobacter infans” in fecal samples of children under the age of 2 in Peru*". We have revised the manuscript according to the comments provided and have provided a response to each below.

Sincerely,

__________________

Margaret N. Kosek, MD

Associate Professor,

Office: (434) 982-6768

E-mail: mnk2n@hscmail.mcc.virginia.edu

**Materials and Methods**

**Reviewer #1:**

Comment 1: With regard to data and result reporting please provide full details when reporting proportions and be more clear in presenting how much out of how much and what. Also it is not that odds of diarrhea are associated with presence of Campylobacter, it is diarrhea itself, and that relationship is expressed through odds ratio. Please be careful in writing of results.

Response 1: Line 235 -237 & Line 400-402: We have edit the sentences for clarity.

Comment 2: Line 229 – what about adjustment for other gene targets?

Response 2: A single copy of *cadF* (*C.jejuni/ C.coli*) and *lxpA* (*C. infans*) is present per genome making this adjustment unnecessary for these genes. This sentence has been added to the manuscript for clarity.

Comment 3: Line 143 – why were these changes to the protocol needed?

Response 3: Line 147. The protocol changes were made to increase the size of library inserts above 500 bp. The manufacturer’s instructions gave inserts below 300 bp. We also have recently changed the library prep kit we use and this change is noted. We have noted the change- “The libraries were prepared using Illumina DNA Prep Tagmentation kit (Illumina, San Diego, CA), following the manufacturer’s instructions except for changes that increased library insert size to a median of 631 bp and a size range between ~375-1100 bp. This was done by decreasing 1st and 2nd volume of Sample Purification Beads to 10 μl and 2.75 μl, respectively. The increase was necessary since standard manufacturer’s instructions resulted in inserts mostly below 300 bp.”

**Results**

**Reviewer #1**

Comment 1: Line 184 – I guess it should be median and not mean.

Response 1: Line 384: We have calculated the median age. Results are corrected.

Comment 2**:** Line 197 – were not significantly higher sounds like a one-tail test was performed, and if it was not why not write not significantly different (includes both higher and lower).

Response 2: Line 400: We have changed the wording of this sentence. A two-sided t-test was used.

Comment 3: It would be helpful in presenting Campylobacter +ve, C. jejuni/coli +ve, C. infans +ve and other species to provide a full cross-tabulated details to follow what are the actual results. What I mean, is data, for instance, how many 16S qPCR +ve samples had +ve results od species-specific qPCRs? Were there any species-specific +ve results that were -ve by 16S qPCR etc. Also, how was actually the prevalence of other, untested, Campylobacter species calculated (provide numerators and denominators).

Response 3: We have added numerators and denominators for every single percentage presented (Line 379-399). None of the *cadF* and *C. infans* positive samples were negative for 16S.
There were only 14 samples tested using metagenomics. Complete results of each specimen are presented in Table 3. We only tested *cadF* and *lpxA* using qPCR. We did not determine the prevalence of other Campylobacter species.

Comment 4: Line 221 – how correlated? were correlation tests used?

Response 4: The pairwise correlation coefficient between the number of reads and infans quantities shown in table 3 is 0.7548. This coefficient was significant at the 5% level. This detail was added to the text (Line 517).

Comment 5: Line 234 – were all mismatches only single mismatches per primer but sometimes single on both primers? You place a lot of emphasis on this to support differences in sensitivity of PCR, this could have easily been checked with degenerate primers in equimolar amounts. Why wasn’t it tried? Why didn’t you test that by mapping your metagenomic reads to cadF? This study was a perfect opportunity to test other primers (hipO, mapA. ceuE.., or modify them to support these claims (at least on smaller subsets).

Response 5: Before this study, we did not realize that the *cadF* qPCR was a problem for the detection of *C jejuni* and *C coli*. The *cadF* primers have been used as a gold standard by many laboratories throughout the world. If our study site was in the US or Europe, we would likely have ordered new primers that would arrive in days. However, in the Peruvian Amazon, this is not the case. Procurement of laboratory items takes weeks or months. Moreover, this study was performed during the COVID pandemic, which further restricted procurement.

We have noted *cadF* mapping reads in the results-Line 539: “Despite the low number of metagenomic reads that mapped to *C jejuni* and *C coli,* four reads in two samples mapped to the *cadF* gene. One read from sample 2678 included the *cadF-*Fw primer-binding site with no mismatches. Unfortunately, this fact does not provide a reason for the negative *cadF* qPCR result.”

**Discussion**

Comment 1: Line 243 – this is wrong. Campylobacter was already recognized as the leading cause before culture-independent methods were being used. You could check history of Campylobacter since late 60’s and early 70’s when finally a method for culturing was devised in Belgium. Everyone in the world started then using it and reporting it as the leading cause…

Response 1: This sentence was referencing the United States not globally, and was written based on FoodNet data provided here: <https://wwwn.cdc.gov/foodnetfast/>. Data from 1996-1998 shows *Campylobacter* with a higher incidence rate than *Salmonella*, but from 2001 - 2012. That said, from 2013 onwards, *Campylobacter* is presented as the food-borne disease with the highest incidence rate. Furthermore, Scallan et al from 2011 a very detailed study of the most common foodborne pathogens in the United States found that utilizing data from 2000 – 2008, *Salmonella* was responsible for 11% of foodborne cases, whereas *Campylobacter* was responsible for 9% in the United States. This reference has also been cited in the manuscript for the sentence (Line 560).

Comment 2: Line 249 – why would this explain in LMIC? If culture is harder to do then molecular methods are used instead? That would imply better sensitivity so no gap….please make clearer.

Response 2: If I understand the question correctly, the detection gap between culture independent techniques and culture is especially pronounced in LMICs settings because non-selective campylobacter culture requires animal blood, specialized media and enriched gases which are not always available to laboratories, in addition to trained microbiologist. Another possible explanation is the presence of atypical Campylobacter species in LMICs, which are especially hard to culture and isolate.

Comment 3: Please do not write results in the discussion. Use it to discuss the data not repeating of results section.

Response 3: Because of the importance of the finding this is being re-emphasized in the discussion. We have made sure these results are also written in the results section (Line 571-573).

Comment 4: In general, the discussion could have been more thorough. There are many studies using variety of culture and culture-independent methods. Many gene targets exist, many other species are also emerging and have various results between studies (C. upsaliensis especially).

Response 4: The main gene target for identifying *Campylobacter* has historically been 16S and *cadF*. Over the past 5 years there has been an increased interest in other minor species of *Campylobacter*. We have discussed the use of shotgun metagenomics for the identification of multiple Campylobacter species in human fecal samples. See Line 739 – 747.

**Tables**

Comment 1: Table 1 – Source not fuente. Also provide literature reference for primers, that is more common form.

Response 1: The word “Fuente” has been replaced for “Source”.

Comment 2: Table 3 – was 98% cutoff used for other species too?

Response 2: The cutoff for all strains was 95%. We have added details to the methods section describing the species detection. We have removed the notation in Table 3.

The Geneious Mapper was run with low sensitivity settings, which only maps reads with <10% mismatch between reads and references. When <50 reads mapped to a particular genome in a sample, all reads were analyzed via BLASTn of NCBI nr/nt database to determine that the reads matched only a single species at >95% identity.

Comment 3: Supplementary figure 1 – the boxplots for C. infans show very clearly a different mean location between asymptomatic and symptomatic children. It is very unusual that t test would return the p value of 0.07. Please check data and test implementation and re-run. If this is not different, I’d like to see raw data and test it myself.

Response 3: The basic data set was available on the initial submission and is still available to the reviewer. We have further added the calculated Mean (log10) Gene Copy Number per Gram of Feces for each target. At the end of this document, you will find the code used to calculate these statistics. Specific test results for C. infans are provided below:

**Conclusions**

**Reviewer #2**

Comment 1: Although the overall number of Campylobacter strains included in the study is adequate, I think the authors cannot conclude that this potential new species (C. infans) has no role in the etiology of diarrhea illness in children. I think more studies are needed in other sites in order to determine its real role.

Response 1: The reviewer is correct in that we cannot say that *C. infans* is not associated with diarrhea in any child in the world. However, we have provided evidence that *C. infans* is not associated with diarrhea in this at the population level in Iquitos. This has been specified in the conclusion (Line 346). The authors consider it would be incorrect to state that we have no information regarding the risk of diarrhea with *C. infans* at all.

**Editorial and Data Presentation Modifications**

**Reviewer #1:**

Comment 1: With regard to data and result reporting please provide full details when reporting proportions and be more clear in presenting how much out of how much and what. Also it is not that odds of diarrhea are associated with presence of Campylobacter, it is diarrhea itself, and that relationship is expressed through odds ratio. Please be careful in writing of results.

Response 1: We have provided numerators and denominators for every proportion. The odds comments has been addressed (See response to comments made by the reviewer in the methods and results sections).

**Summary and General Comments**

**Reviewer #1:**

Comment 1: The authors report a diagnostic cross-sectional study of Campylobacter spp.in children in Peru . Molecular techniques have been used for detection and the authors investigated primarily the role in gastrointestinal illness of an emerging Campylobacter species proposed as C. infans. The results showed it is not associated with symptomatic illness and that there is more unaccounted Campylobacter positive infections based on 16S rDNA PCR and metagenomic analyses that are negative by species-specific qPCR used in the study. This is a valuable study for epidemiology of GI pathogens in low income settings and will be of interest to the readership.

With regard to the language and writing, an overall remark is that writing style and format needs to be consistent e.g., writing of numbers at the beginning of the sentence, usage of symbols (%). There are a lot of instances of unnecessary long and convoluted sentences that could be shorter, clearer and more concise. I would suggest one native english speaker to improve the manuscript.

Response 1: We have reviewed the manuscript for clarity throughout.

Comment 2: Keywords – perhaps to change Iquitos to Peru? Could be a better option in literature search.

Response 2: We have added Peru as a keyword.

*Abstract*

Comment 3: Line 30 – investigated instead of measured.

Response 3: “Determined” was used instead of “measured”.

Comment 4: Lines 36-38 – rewrite clearer and shorter. e.g. C. infans presence was not significantly associated with symptomatic illness. Use space gained to perhaps add data on the sample population (age, gender?) or add data on other Campylobacter species detected…

Response 4: The abstract has been reviewed for clarity.

*Introduction*

Comment 5: Line 50 – so, where is the disease highest? And that, as written, is not evidence of the discrepancy between culture and culture-independent diagnostic methods. Rewrite as it is confusing.

Response 5: We thank the reviewer and have revised this sentence for clarity.

Comment 6: Line 53 – where is second reason written? Is it the emerging Campylobacter species?

Response 6: Word change in line 57

Comment 7: Line 68 – which unidentified species, mapped how and to what? Then, similarity in what? Provide details.

Comment 8: Line 67 and 73 – do not report % only data, provide out of how many sequences, samples…

Response 7 and 8: We greatly appreciate this comment since it allowed us to rewrite this portion of the introduction to correct mistakes and add clarity for the reader. The reads were not used to map on an unidentified genome but were used to assemble a nearly complete *Campylobacter infans* genome. The revision is shown below.

Line 98: “*Campylobacter infans* was first described in fecal samples of symptomatic and asymptomatic children under 1 year of age enrolled in the Global Enteric Multicenter Study (GEMS)^13^. This potential new species was identified from a single fecal sample associated with an infant experiencing prolonged diarrhea where *Campylobacter sp.* represented >83% of the fecal microbiome. Through assembly of shotgun metagenomic sequencing reads from this fecal sample, 75 contigs consisting of ~1.7 mb were described as *C. infans* showing less than 75% similarity to the genomes of all other recognized *Campylobacter* species.”

**Reviewer #2:**

This is an interesting study on Campylobacter, evaluating the relevance of a potential new Campylobacter species (C. infans) in children from the Peruvian Amazon. The study includes qPCR and metagenomics studies.

Comment 1: Although the overall number of Campylobacter strains included in the study is adequate, I think the authors cannot conclude that this potential new species (C. infans) has no role in the etiology of diarrhea illness in children. I think more studies are needed in other sites in order to determine its real role.

Response 1: Please see response to the same comment made in the conclusion section above.

Comment 2: In the method and results sections, it is not clear why the authors performed studies on saliva samples. It is on the discussion section that the authors explain the reason of these studies (potential presence of C. infans in the oral cavity similar to C. concisus). This should be mentioned early on the text.

Response 2: We have included the reason for the evaluation of saliva samples in the methods section (Line 196-198)

Comment 3: A very relevant result of the study is the evaluation of the *cadF* gene for the detection of C. jejuni and C. coli. This should be part of the title.

Response 3: This study was not designed to evaluate the performance of the *cadF* primer and probe set. Although we agree that the findings are important, their performance was incompletely evaluated in this report. A full evaluation as mentioned by reviewers would also include the evaluation of other highly conserved sequenced such as *hipO* and *mapA*.

*Stata Code:*

gen eq_m_infans = .

replace eq_m_infans = -3.42

gen eq_m_16s = .

replace eq_m_16s = -3.39

gen eq_m_ipah = .

replace eq_m_ipah = -3.45

gen eq_m_cadf = .

replace eq_m_cadf = -3.17

gen eq_c_infans = .

replace eq_c_infans = 47.32

gen eq_c_16s = .

replace eq_c_16s = 43.23

gen eq_c_ipah = .

replace eq_c_ipah = 41.58

gen eq_c_cadf = .

replace eq_c_cadf = 48.09

gen campy16s_quantlog = ((campy16s_ct - eq_c_16s)/eq_m_16s)

gen cadf_quantlog = ((cadf_ct - eq_c_cadf)/eq_m_cadf)

gen infans_quantlog = ((infans_ct - eq_c_infans)/eq_m_infans)

gen ipah_quantlog = ((ipah_ct - eq_c_ipah)/eq_m_ipah)

gen campy16s_quant = (10^campy16s_quantlog)/3

gen cadf_quant = 10^cadf_quantlog

gen infans_quant = 10^infans_quantlog

gen ipah_quant = 10^ipah_quantlog

gen campy16s_quant_gram = campy16s_quant*1000

gen cadf_quant_gram = cadf_quant*1000

gen infans_quant_gram = infans_quant*1000

gen ipah_quant_gram = ipah_quant*1000

gen campy16s_quantlog2 =log10(campy16s_quant_gram)

gen cadf_quantlog2 = log10(cadf_quant_gram)

gen infans_quantlog2 = log10(infans_quant_gram)

gen ipah_quantlog2 = log10(ipah_quant_gram)

ttest campy16s_quantlog2 , by(outcome)

ttest cadf_quantlog2 , by(outcome)

ttest infans_quantlog2 , by(outcome)
